# Motivation to Participate in Intergenerational Programs: A Comparison across Different Program Types and Generations

**DOI:** 10.3390/ijerph19063554

**Published:** 2022-03-17

**Authors:** Jiska Cohen-Mansfield

**Affiliations:** 1 Minerva Center for Interdisciplinary Study of End of Life, Tel Aviv University, Tel Aviv 6139001, Israel; jiska@tauex.tau.ac.il; Tel.: +972-3-6407337; Fax: +972-3-6407339; 2 Department of Health Promotion, School of Public Health, Sackler Faculty of Medicine, Tel Aviv University, Tel Aviv 6139001, Israel; 3 The Herczeg Institute on Aging, Tel Aviv University, Tel Aviv 6139001, Israel; 4 Igor Orenstein Chair for the Study of Geriatrics, Tel Aviv University, P.O. Box 39040, Ramat Aviv, Tel Aviv 6139001, Israel

**Keywords:** intergenerational programs, community-based programs, older adults, young adults, intergenerational relations, intergenerational program evaluation, motivation

## Abstract

Much research has attested to the benefits of intergenerational programs (IGPs) for older and younger participants, but there is a lack of understanding about what motivates them to participate and to persevere. We conducted structured interviews with 83 older (mean age = 77) and 96 younger (mean age = 23) participants who participated in 13 IGPs in Israel, some involving specific topics, and some providing assistance to older adults. Using a mixed-methods approach, we analyzed differences in motivation across generations and program types and compared initial and ongoing motivation to participate. We found differences regarding motivation by age group and program type: Among older participants, interest in the specific subject was a more prevalent motivation in topic-focused groups, while receiving support was more common in assistance groups. Among young persons, motivations relating to obligation, such as receipt of a financial scholarship, and the wish to help others were the most prevalent motivators. Ongoing motivation was often explained by positive intergenerational relationships and enjoyment. For older adults, offering more diverse topic-focused activities may motivate greater participation. For young adults, integrating IGPs within more and different settings, and promoting IGPs as opportunities to help others are potential motivators.

## 1. Introduction

Intergenerational programs [IGPs] are types of human services that involve ongoing and organized interactions between members of younger and older age groups for the benefit of all participants [1]. They are typically established to strengthen social cohesion, create links between generations, and encourage creative community activity. The goals of IGPs are to increase contact and understanding, create meaningful relationships, promote emotional and social growth, and achieve various educational or community goals [2,3,4]. 

The negative physical and mental health impact of social isolation and loneliness on older adults has been widely studied [5,6,7], yet young people also struggle with these issues [8,9,10]. IGPs can address social isolation and loneliness for both generations [11]. IGPs are designed to create positive relationships between generations [12], reduce negative perceptions of the other generation [13], improve physical and mental health outcomes [14,15], decrease loneliness [16], and increase social connection [17]. Peacock and O’Quin (2006) [18] claimed that bridging generational gaps through IGPs “can … foster the development of new roles, and provide purpose and meaning in a life stage where limited opportunities for such may exist” (p. 368). 

Research has also identified challenges, including uneasiness experienced by young people when encountering older people with dementia or frail older adults [19]. Another study found that when older adults encounter environments that are not age-appropriate for them, they may disengage from participation [20]. 

Although the motivation of participants to take part in IGPs has not been studied specifically, several studies have mentioned the willingness of participants to continue participating in IGPs, e.g., [21]. 

In one study, university faculty members worked in collaboration with a team of older volunteers to develop, deliver, and evaluate an undergraduate intergenerational course about aging [22]. The opportunity to contribute was the most salient motivation for joining the collaborative team. Some participants wanted to help the younger generation by “challenging their assumptions” about older people and broadening students’ life experiences. Some older volunteers explained that participating was fun, or that they felt validated because the students valued their participation. In a follow-up report, the researchers indicated that other participants felt that volunteering helped them remain cognitively active [23]. In a different study involving older volunteers who mentored children in urban public elementary schools, the volunteers described a range of motivational factors from altruism to self-interest [24]. 

The limited available literature principally describes the motivation of older adults, who generally report more benefits than young adults in IGPs [25]. Little research has been conducted on the motivation of young participants since most studies involved school students whose participation was not elective [21].

In this paper, we aim to address the lacuna in the research regarding the motivation of younger and older individuals to participate in IGPs. Addressing this gap aims to provide organizers of IGPs with new insight into the recruitment and retention of participants. Our research questions are: (1) What motivates young and older participants to participate in IGPs? We examined both initial motivation and ongoing motivation for both groups. (2) How do the different types of motivation relate to young and older participants’ background characteristics? (3) For young participants, we also examined whether they perceived their involvement as voluntary or obligatory.

## 2. Methods

### 2.1. Participants and Recruitment

We sought to include all the types of community-based IGPs offered in the greater Tel Aviv, Israel metropolitan area by contacting national and municipal offices and diverse not-for-profit organizations. We learned of additional programs through snowballing. Our data were generated from 13 programs. We included only IGP programs that were organized by community-based organizations, in contrast to school-based, and were conducted at community centers or participants’ homes, rather than in school buildings or long-term care settings.

We categorized the 13 community-based IGPs found in greater Tel Aviv based on their most salient content and goals: (1) “Topic-focused programs,” which involved the performing arts such as theater and dance, the study of Jewish texts, a photography course, and a bridge club; (2) “Assistance groups” designed to provide one-on-one support with daily activities for older persons, including visits to their homes, documentation of their life stories, in-home computer assistance, or assistance with pet care. Young participants and older participants met about once weekly on average. 

While all programs were aimed at improving intergenerational relationships, the topic-focused programs provided meaningful recreation rather than personal assistance. Some programs recruited young persons from among specific groups, e.g., university students, soldiers. Young participants either volunteered or participated non-electively as part of their military, pre-military, national service, or academic course work. Some young participants received a financial or housing scholarship for their participation. Older participants were referred to some IGPs by social services workers, or by word of mouth, or responded to publicity about opportunities to participate in such programs.

### 2.2. Procedures

The study was approved by the ethics committee of Tel Aviv University. After informed written consent was obtained, questionnaires were either completed during personal interviews by trained interviewers (~80%) or were self-administered.

### 2.3. Assessments

The questionnaires first inquired into participants’ demographic background. The subjective health of older participants was measured using the global self-rated-health item on SF-36 [26], using a scale from 1 = bad to 5 = Excellent.

Motivation for participation in the IGP was queried via three questions:

- Why did you choose to participate in the activity?
- What motivates you to continue to take part in the activity and persevere in it?
- Is the activity a mandatory or voluntary task for you?

The third question was addressed only to young participants. While the last question requested a binary (Yes/No) response, all young participants were also asked to specify the elective or non-elective nature of their involvement. These clarifications and responses to open-ended questions enabled us to identify the organizational framework within which 91 (out of 96) young participants participated in the IGPs. 

### 2.4. Analytical Approach 

This study used a mixed-methods approach, combining quantitative and qualitative analyses. The research question as to what motivated young persons and older persons to participate in IGPs was first examined qualitatively. We then used the themes reached to code the responses of each participant. We used descriptive analyses of frequencies and cross-tabulation to respond to the research questions.

Qualitative analysis. We used a thematic approach to understand participants’ motivations for participating in IGPs [27]. To adhere to the criterion of reliability [28], which, in qualitative research, refers to the stability of responses to multiple coders of the data, four research staff members were involved in reading and organizing the data and in deliberations on the labeling and selection of themes. Initially, two different research staff members read the narratives provided in response to the questions about motivation. Each staff member independently classified and coded the narratives. They then discussed consistencies and discrepancies and revised accordingly. For those topics concerning which there were disagreements, two other research staff members read the narratives and coded the responses, after which they too discussed the consistencies and discrepancies. To obtain a sense of the degree to which various themes emerged, we calculated their frequencies and determined the percentages of participants from whom the respective themes emerged.

Quantitative analysis. We used chi-square and *t*-tests to examine the relationships between background information and motivational factors. Because of the exploratory nature of those analyses and the use of multiple comparisons, we applied a criterion of *p*-value of 0.001 for presentation of significant findings. 

## 3. Results

### 3.1. Participants’ Background Characteristics

We interviewed 83 older persons and 96 young persons. For older participants, the mean age was 77.07 (SD = 7.07), and 83.1% were women. Of the older participants, 46 (55.4%) participated in the topic-focused groups and the rest in the assistance groups. Young participants reported a mean age of 23.49 (SD = 6.25), and 66.7% were women. Of the young participants, 32 (33.3%) participated in the topic-focused groups and the rest in the assistance groups. Age and gender were not significantly associated with type of IGP in either age group. Older participants in the topic-focused IGPs reported better health (mean = 3.42) than those in the assistance groups (mean = 2.67, t_79_ = 3.34, *p* = 0.001).

### 3.2. Sources of Motivation

Concerning what motivates young persons and older persons to participate in IGPs, we separately coded the questions about initial and ongoing motivation to participate. The resulting frequencies of various motivating factors are presented in Table 1 and are illustrated by quotes below. Each theme is illustrated by a quote, which is then identified by the participant’s study-assigned number, sex, age, type of activity, and whether the quote referred to initial motivation (IM) or ongoing motivation (OM). When original quotes used names, a pseudo name of the same sex was used.

### 3.3. Older Participants

Interest in the subject matter, IM and OM. “[I came] because of the subject of the course—photography; It filled a few extra hours for me” (#55-M-74-Topic-Focused (TF)-IM) or “I really like theater and the subject is very interesting for me. When I was young, I participated in a number of plays and then I did not [further] develop this [interest]. And it intrigued me and I wanted to try and see how it would go” (#84-F-80-TF-IM) or “It is relaxing and good. This photography is relaxing and there are beautiful things to photograph in this country” (#25-F-70-TF-OM).

Satisfaction and joy, OM. “I enjoy every moment, a great pleasure” (#198-F-76-TF-OM) or “The activity gives me pleasure” (#176-F-78-TF-OM). “The joy I have in my heart, I love it. It’s love” (#87-F-79-TF-OM).

Intergenerational interactions and friendship, IM and OM. “They give me meaning in life, content. I am getting to know these wonderful youth” (#143-F-90-assistance-IM) or “It interests me to be among the youth” (#184-F-77-TF-IM) or “I love being close to the younger generation, I feel young in spirit and love to dance” (#197-F-78-TF-IM) or “The fact that it gives me pleasure, I feel with them … they opened up to me. I tell about my life and they tell me about their life, they are like my granddaughters. I feel like I have expanded the family” (#171-F-77-assistance-OM).

Receiving support (e.g., help, company), IM and OM. “I asked for someone to come to me because I am alone. There are afternoons when I don’t even have anyone to talk to. I wanted someone to come so that I will have someone to talk to and who will help me with all sorts of things I need” (#142-M-83-assistance-IM) or “I wanted someone to teach me [about] a computer. I have a computer and did not know how to use it. When the kids were little, I avoided the computer and now I really like this device and just want to know how to develop [using] it. Bringing someone in costs a lot of money; [A volunteer organization] had a course and they stopped it … the best thing is to have someone come and sit by you and guide you, that’s the best” (#44-F-75-assistance-IM) or “It gives me a lot, I am very satisfied. I always say, [when] Ron returns he is the best. I love him, it’s not like a woman’s love, it’s like a mom and son’s love. He does everything for me when he can. He helps me a lot. I give him a score of one hundred” (#17-F-85-assistance-OM).

Invited to participate, IM. “Rachel asked me to attend, so I agreed. I do not know the specific neighborhood, so it was interesting for me. It’s nice to be with young people; They instructed us nicely” (#30-F-80-TF-IM) or “I did not choose [the IGP], my friend, Rhonda, who works here at the club, teaches gymnastics or something here, told me that there is this activity and I said I would come and check it out” (#99-F-74-TF-IM).

Activity/Having something to do, IM and OM. “Ever since I retired, against my will, I started looking for what to do. To my great joy, I received a gift from the job from which I had been fired, a personal computer, and I said to myself, what am I doing now?! What do I do in life? Then I started writing my life story. It took me 10 years. After that, I started writing short stories and participated in a creative writing class. And then I did research on a family that perished in the Holocaust; No one knew about the others’ existence, and finally the second generation found each other. It’s a life story of this family. So now I’m looking for activities, and each time a different activity. …Then I heard about the community theater, and I said, ‘Oh!’ Now I have something to do” (#89-M-77-TF-IM) or “A friend told me it exists; It’s something I’ve never done before. I have time now, something I have never had in my life. So, I came, I had a nice time, so I stayed. Everything was so light. Without major goals. Had it not been interesting, I would have left, but it was fine, so I stayed” (#104-F-70-TF-IM) or “Because [otherwise] it’s a day with no activities” (#81-F-79-TF-OM).

Sense of commitment, OM. “It’s a responsibility, as soon as I get into something, then I take it upon myself to complete it. I take on a new challenge every year, and every time I take on such a challenge I keep going until the end. I intentionally take on all sorts of things every year that present a challenge for me “(#79-F-71-TF-OM) or “It’s a matter of character; I will not leave in the middle…” (#92-F-70-TF-OM).

Philanthropy/Desire to help young persons, IM and OM. “Because it fulfills me, it’s my life. It is important for me to pass on to the young people the values with which I grew up” (#36-F-82-assistance-IM) or “It is my pleasure that I can teach young people and pass my knowledge on to them” (#36-F-82-assistance-OM).

### 3.4. Young Participants

Obligation, reported both as IM and OM. “Obligation” refers to young participants’ reports that IGP participation was a non-elective activity. “[I participated] because it is mandatory to make a personal commitment and this activity was the most convenient and appropriate for my schedule” (#199-F-16-TF-IM). The obligation may have been part of national service enlistment, army service, in those units that require it, or as a condition for receipt of a financial study or housing scholarship, etc. “The military required that we participate” (#7-M-21-assistance-IM) or “I committed to the activity at the beginning of the year so I persevered with it” (#199-F-16-TF-OM).

Interest in the subject matter, IM and OM. “I have been participating in community theater for three years in a row in this course. There are quite a few frustrations and challenges in this … but I do it because it is the only production in the theater department that allows all students to participate in a creative way, and writing is also one of the things that interests me and this is the only course that allows one to write. Plus, it allows me to perform on stage” (#111-F-25-TF-IM) or “I was interested in learning bridge” (#185-M-16- TF-IM) or “Both the stories themselves and the enthusiasm of the older women motivate me to continue” (#34-M-19-assistance-OM).

Philanthropy/Helping others, IM and OM. “It was important for me to do something for the community before I graduate from university” (#52-M-27-assistance-IM) or “[I liked] the idea of a year of contribution to the State, in the framework of a year of national service, the understanding that I am helping and really contribute where I am needed” (#137-F-19-assistance-OM).

Satisfaction with the activity and enjoyment of the activity, OM. “Personal satisfaction” (#7-M-21-assistance-OM) or “I really, really enjoy it …. love to come to meetings with Hanna” (#51-F-19-assistance-OM).

Intergenerational interactions and friendship, IM and OM. “I chose to take part in the activity because I also believe in the goals of the activity; I think it is important to get the generations closer. In addition, the older generation has a lot to contribute to us, the younger generation. There is something to learn from them” (#203-F-16-TF-IM) or “I connected to that woman and that moving life story of hers, and also the need to feel the same feeling of satisfaction when I walk away knowing I made her smile and enjoy those moments” (#133-F-24-assistance-OM).

Personal or professional value/experience, IM and OM. “I worked at [another volunteering organization that provides a scholarship] for three years, and I wanted to diversify a little for myself. I got a lot out of it, I do not shy away from older persons, like many people who flinch” (#154-F-26-assistance-IM) or “For me it’s part of personal development … it gives me answers and questions about my life…it connects me to the world I study, the whole subject of coaching. It gives [or] connects me to the spiritual side of what I am learning. Gives me a perspective that is more human, in the Jewish point of view and less religious … It is more a matter of a humane, philosophical outlook. This is a field that interests me very much” (#80-M-44-TF-OM) or “I think I derive something from it … I’m getting to know a population that I hadn’t had much contact with. It’s seeing different perspectives. I guess it’s also somewhere nice to have another circle of friends, both from my side and from hers” (#151-M-28-assistance-OM).

Sense of commitment, OM. “I began the activity so I will finish it. It is not fair to stop in the middle, also to the old woman” (#60-F-20-assistance-OM) or “Commitment to the group, desire to learn to direct and gain experience” (#124-F-31-TF-OM).

### 3.5. Comparing the Motivation of Older and Young Participants

Overall, the most common motivational factors that prompted older participants to become involved in IGPs (Table 1) were interest in the subject matter, being invited to participate, and receiving support. Ongoing motivation was based on satisfaction derived from the activity, continuing interest in the subject matter, and favorable intergenerational interactions and friendship. In contrast, the most common factors that motivated young participants to become involved were service, school, or financial requirements, followed by the desire for intergenerational interactions and interest in the subject matter. Ongoing motivation was based on favorable intergenerational interactions and friendship, and satisfaction and joy derived from the activity. Overall, young participants tended to mention more motivational factors than older participants (t_177_ = 4.04, *p* < 0.001, see Table 1).

### 3.6. Voluntary vs. Mandatory Participation of Young Persons

To understand the extent to which young participants’ involvement was actually voluntary or mandatory, we cross-tabulated the organizational frameworks (service, school, financial) under which the young participants participated by their reports of the activity being voluntary or mandatory—both in the open-ended questions about their motivation to volunteer and in a question that directly addressed whether their activity was voluntary or mandatory. As shown in Table 2, only 10 young participants (around 11%) were actual volunteers in the sense that they were free of any requirement to participate. The vast majority were obligated to participate, although a large proportion of those reported that the activity was voluntary. 

### 3.7. The Relationships between Sources of Motivation and Background Variables among Young and Older Participants

Since the examination of the relationships between reported sources of motivation and background variables was exploratory and involved multiple comparisons, only relationships that were found to be significant at a level of *p* ≤ 0.001 are reported.

Age, gender, and health. Older participants who reported interest in the topic as a motivational source were younger than older participants who did not report such interest (mean ages 74.5 vs. 79.6, t_79_ = 3.45, *p* = 0.001). Similarly, older participants who reported sense of commitment as a motivational source were younger than older participants who did not report so (mean ages 71.2 vs. 77.4, t_15_ = 4.99, *p* < 0.001). Older persons who reported receiving support as a motivation for participation reported worse health status than those who did not (mean health status 2.4 vs. 3.4 t_79_ = 4.20, *p* < 0.001). No relationships with gender fit our criteria. For young participants, none of the relationships fit our criteria. 

Type of activity. Among older participants, those who were in the assistance groups were more likely (70.3%) to report that the activity was motivated by need for support and companionship as compared to 2.2% in the topic-focused groups (χ^2^_1_ = 43.3, *p* < 0.001). Conversely, older participants in the topic-focused IGPs were more likely to report interest in the topic as their motivation (73.9%) as compared to those in the assistance group (18.9%, χ^2^_1_ = 24.81, *p* < 0.001). Among young persons, those who were in the assistance groups were more likely (56.3%) to report that the activity was motivated by philanthropy (altruism) as compared to those in the topic-focused groups (21.9%, χ^2^_1_ = 10.19, *p* = 0.001).

### 3.8. Organizational Framework through Which Young Persons Participated

We compared young persons’ reports of motivation in terms of the four organizational frameworks through which they participated: (1) pre-military, military, or national service, *n* = 35, (2) school project, academic course, *n* = 23, (3) condition for financial scholarship, *n* = 23, and (4) purely voluntary activity not related to a formal framework, *n* = 10. As summarized in Table 2, among those receiving financial scholarships, 78.3% indicated in the qualitative analysis that their IGP participation was mandatory, as compared to 68.6% of those enlisted in pre-military, military, or national service programs that reported their participation was mandatory, whereas a lower percentage of young participants reported their participation as mandatory when it was part of a school activity (26.1%) or those who were actual volunteers (0%, χ^2^_3_ = 27.25, *p* < 0.001). Philanthropy/helping others was often a motivation reported by actual volunteers (80%) and by those providing a year of military, pre-military, or national service (63%), as compared to those receiving a financial scholarship (34.8%) or those in a school-related activity (13.0%, χ^2^_3_ = 19.92, *p* < 0.001). Interest in the subject was most often mentioned by actual volunteers (80%) and by those in school-related activities (60.9%), but much less so among those receiving financial scholarships (17.4%) or those enlisted in military, pre-military, or national service (17.1%, χ^2^_3_ = 23.66, *p* < 0.001). 

## 4. Discussion

The most striking finding was that about 90% of the young participants were in educational or enlisted-service frameworks that mandated “voluntary” activity, either in IGPs or in other endeavors. This is especially illuminating in view of the fact that we located the young participants not through those educational or occupational frameworks but through the IGPs. This suggests that the vast majority of young persons in IGPs, at least those in the geographical area under study, are not truly volunteers. This finding corroborates previously reported complaints by organizers of IGPs concerning the difficulty of recruiting young persons [29]. Our results also suggest that a vast majority of young persons are not sufficiently self-motivated to participate in IGPs without additional inducement. Rather, service-required, academic-required, or financial scholarship-required frameworks appear necessary to direct young persons to participate in IGPs. This points to a limiting factor in developing IGPs in that the pool of young volunteers is insufficient without monetary incentives or the imposition of service obligations. 

The distinction between “obligatory” and “voluntary”, however, is somewhat obscure in this context. Over half of the young participants belonging either to a pre-military, military, or national service organization, and many of those receiving a financial scholarship that required participation, described their involvement as “voluntary.” This is probably due to two types of reasoning. The first is that the choice of enrolling in the organizational framework is usually voluntary, i.e., participants choose to take the specific course or to apply for the financial scholarship that comes with conditions; the second, reported in some of the verbal responses to our open-ended questions, is that participants have the option to choose from several “voluntary” activity options. Participants who felt that they “chose” to enlist in national service, or “chose” the academic course that required participation, or “chose” the particular IGP from a number of options, considered their participation voluntary.

Among older persons in the assistance programs, 70% said they were motivated by a need for support and companionship. In a sense, individuals among these older persons were compelled to participate because they required help and/or were lonely, and the IGP was the only available solution. In addition, close to 30% of all older participants said they joined because they were invited to attend, suggesting that observers perceived a need for intervention, and 10% said they enrolled because it gave them something to do, suggesting a need for occupation. Similarly, the findings point to significant relationships between the background variables of age and health status and the source of motivation to participate in IGPs. Thus, for some older persons, involvement in an IGP, while not mandatory, was in some way dictated by their life circumstances.

Reported motivational factors differed between young and older participants in several ways. The sources of motivation of “obligation”, ”philanthropy” (altruism), and “valuable experience” (for personal or professional growth) were mentioned mostly by young participants. In contrast, the factors of “being invited to participate”, “receiving support” and “having something to do” were mentioned only by older participants. Some motivational factors were reported by both young and older participants, including “interest in the subject matter”, “satisfaction and joy derived” (or anticipated) from the activity, “intergenerational interactions and friendship”, and “sense of commitment.” The differences we found partly reflect the different life experiences of the respective generations. For example, older, but not young participants, reported the need for support and the need to fill available time. The differences also reflect that a large proportion of the IGPs under study were devoted to providing assistance to older persons, which relates directly to the altruistic motivations expressed among young participants and the need for support expressed among older participants. For older participants who were involved in the topic-focused groups, interest in the subject matter was often a key motivation. 

We found that motivational factors change with time during the course of IGPs. The motivations of satisfaction and joy, as well as intergenerational interactions and friendship developed into ongoing motivations as the programs continued, whereas other motivational factors, such as interest in the subject matter (among older and young persons) and personal/professional value (among young persons) tended to decrease. To some extent, this suggests that the success of the IGPs in our study provided the motivation needed to promote continued participation. The implications of the motivational factors that emerged in this study should be investigated in future studies. For example, were young persons who cited enjoyment of intergenerational interactions and friendship as ongoing motivations to participate in IGPs more likely to manifest a decrease in ageist attitudes or an increase in interest in pursuing a career involving older adults? Were older persons who cited enjoyment of intergenerational interactions and friendship as ongoing motivations more likely to report a decrease in feelings of isolation and loneliness? As both young and older participants cited “interest in the topic” as a motivating factor, offering more diverse topic-focused activities may motivate greater participation, in addition to integrating IGPs within more and different settings, and promoting IGPs as opportunities to help others.

A study of an American IGP, “The Experience Corps” [24,30], in which older adults mentored urban elementary school children, offers relevant results concerning sources of motivation to participate in IGPs. This IGP, while aiming to help children, in fact benefitted both the program’s older participants and the children [24,30]. The study reported the following sources of motivation among older participants: altruism (about 60%), ideology (~65%), material reward (~30%), status reward (~10%), social relationships (~20%), use of spare leisure time (~40%), and personal growth (~25%) [24]. Our findings echo many of these motivational factors, but the relative weight of the factors is divergent because of differences in the content of the U.S. program vs. the topic-focused and assistance-focused IGPs we studied, and because of the differing characteristics of the respective programs’ older participants. In contrast to the Israeli IGPs we studied, in the case of the U.S. IGP, it was older persons who received financial incentives, and these were considered essential for recruitment and retention [24]. The cost per volunteer was reported to be USD 3613 in 2004 [30]. 

We aimed to recruit all IGPs in the greater Tel Aviv metropolitan area. Therefore, the study is limited to programs in the cultural context of Israel. We also limited the IGPs to those in the community, rather than in schools. As participants volunteered to participate in the research, the data may represent a bias towards more successful experiences in IGPs. Another limitation is the use of qualitative analysis to determine the distribution of sources of motivation, whereas future research should validate those findings via quantitative questionnaires based on our findings, which are likely to provide more precise estimates. These limitations notwithstanding, this study is innovative in examining in depth young persons’ motivation to participate in IGPs, in examining the complex meaning of “volunteering” in this context, in comparing young and older persons’ motivations to participate, in studying how those motivational factors change during the course of IGPs, and in examining community-based rather than school-based IGPs. The study is strengthened by convergent results vis a vis the few studies available on the topic of motivation to participate in IGPs and by its use of a larger sample than most studies of IGPs, thus offering valuable guidance for future planning of IGPs. 

## 5. Conclusions

The findings highlight the crucial need for motivational analysis prior to initiating an IGP. In our study, the often-proclaimed virtues of IGPs, such as promotion of intergenerational understanding and encouragement of altruistic community involvement, emerged as insufficient without the weight of additional self-interest or external requirements as motivational factors. Most topic-focused programs provided some self-interest in the form of offering an experience that would further knowledge or experience in a particular topic, but among such IGPs, most young participants were bound by additional obligatory frameworks. In the assistance groups, the self-interest of older participants was evident, and although more than half of young participants in the assistance groups reported philanthropy/altruism as a motivational factor, it is likely that many would not have participated without having been obligated to do so. These findings are important because they challenge the general perception that recruiting “volunteers” is an inexpensive means of improving intergenerational relationships and remedying social problems. Rather, the results suggest that significant funding or embedding IGPs in a compulsory framework may be needed. The motivational factors of “satisfaction”, “interpersonal relationships”, “idealism/sense of philanthropy”, and “interest in the topic” appear to need a boost from a different realm. These complexities need to be considered in planning IGPs and evaluating their outcomes. Future research should examine correlations between the motivations of dyads in one-on-one IGPs and within specific topic-based groups. Future work could also include a cost/benefit analysis of IGPs by following cohorts on a longitudinal basis, exploring participants’ ongoing and changing sources of motivation, and also the long-range impact on participants, such as the percentage of young participants who go on to pursue careers involving older adults.

## Figures and Tables

**Table 1:** Frequency of different self-reported motivational factors by initial vs. ongoing motivation and by young persons and older persons.

Motivational Factor		Older Persons (*n* = 83)			Young Persons (*n* = 96)		
		IM	OM	Total	IM	OM	Total
Obligation					42.7%	19.8%	50%
Interest in the subject matter		45.8%	32.5%	49.4%	31.3%	13.5%	35.4%
Philanthropy/Helping others		1.2%	2.4%	2.4%	24%	28.1%	44.8%
Satisfaction and joy		1.2%	44.6%	44.6%		34.4%	34.4%
Intergenerational interactions and friendship		15.7%	22.9%	36.1% ^a^	32.3%	37.5%	55.2% ^a^
Receiving support (e.g., help, company)		28.9%	18.1%	32.5%			
Personal or professional value/Experience					25%	8.3%	29.2%
Invited to participate		28.9%		28.9%			
Activity/Having something to do		6%	4.8%	9.6%			
Sense of commitment			7.2%	7.2%		15.6%	15.6%
Number of Motivational Factors per person	Range	1–3	0–3	1–4	0–4	1–4	1–5
	Mean	1.28	1.33	2.11 ^b^	1.55	1.57	2.65 ^b^
	SD	0.53	0.57	0.83	0.69	0.71	0.95
	Median	1	1	2	1	1	3

Comparing older vs. young participants’ results: ^a^ χ^2^_1_ = 6.51, *p* = 0.01; ^b^ t_177_ = 4.04, *p* < 0.001.

**Table 2:** The comparison of the organizational framework of the activity and the mandatory/voluntary perception of young persons participating in IGPs.

	Participation Perceived as		
Question	Mandatory/Voluntary (*n* = 83)		Qualitative Analysis Indicating Obligation Motivation (*n* = 91)
Organizational Affiliation of Young Participants	Voluntary	Mandatory	
Pre/military/national service (*n* = 32–35)	56.3%	43.8%	68.6%
School project/Part of studies (*n* = 19–23)	31.6%	68.4%	26.1%
Scholarship (Financial) (*n* = 22–23)	59.1%	40.9%	78.3%
Personal voluntary activity (*n* = 10)	100%	0%	0%

## Data Availability

Qualitative data are not available due to confidentiality concerns. Quantitative data may be available upon request directed to the author, contingent upon approval from the IRB of Tel Aviv University.
